# *Science and sanity*: A social epistemology of misinformation, disinformation, and the limits of knowledge

**DOI:** 10.1177/13634615241296301

**Published:** 2024-11-26

**Authors:** Laurence J. Kirmayer

**Affiliations:** Division of Social & Transcultural Psychiatry, McGill University, Montreal, Canada

**Keywords:** social epistemology, ways of knowing, cognitive biases, epistemic communities, social media, misinformation, disinformation, delusions, conspiracy theories, science education

## Abstract

Recent challenges to scientific authority in relation to the COVID pandemic, climate change, and the proliferation of conspiracy theories raise questions about the nature of knowledge and conviction. This article considers problems of social epistemology that are central to current predicaments about popular or public knowledge and the status of science. From the perspective of social epistemology, knowing and believing are not simply individual cognitive processes but based on participation in social systems, networks, and niches. As such, knowledge and conviction can be understood in terms of the dynamics of epistemic communities, which create specific forms of authority, norms, and practices that include styles of reasoning, habits of thought and modes of legitimation. Efforts to understand the dynamics of delusion and pathological conviction have something useful to teach us about our vulnerability as knowers and believers. However, this individual psychological account needs to be supplemented with a broader social view of the politics of knowledge that can inform efforts to create a healthy information ecology and strengthen the civil institutions that allow us to ground our action in well-informed picture of the world oriented toward mutual recognition, respect, diversity, and coexistence.

You’re confounded by leaders as authentic as Princess Caraboo and the scrap metal Lustig sold as the Eiffel Tower. Oh, how they chant their chants: *War is peace*, *freedom is slavery*—and most importantly—*ignorance is strength*. That's right: the less you know, the more indignant. The less you know, the more protected. The chorus is no less than: Say it loud, I’m here and I’m proud. Doesn’t that sound familiar? Take a breath. Let the chorus go silent, walk away on a path of your own design and determination, even if you lose your way a few times during the journey. Don’t be afraid of the loneliness. (Obejas, 2021, p. 55)

This breathless epigraph comes from a prose-poem by the Cuban-American writer Achy Obejas that speaks to the crisis of truth in our time: impostors everywhere, *narishkeit*—a Yiddish word that means nonsense, foolishness or sheer stupidity—on heavy rotation, and knowledge foraging on the internet devolved to become a kind of epistemic dumpster diving. Though some of the concern with misinformation may be exaggerated (Altay et al., 2023), deliberate efforts to undermine the news media and scientific expertise are threatening the institutions of civil society (Adler & Drieschova, 2021). The problems we have with truth have a long lineage, but their current form is largely due to the deliberate exploitation of new social media to advance commercial or political agendas. This has prompted efforts to develop the discipline of agnotology, studying how ignorance is actively produced to serve the interests of corporations or others in power, creating confusion about basic facts, impeding policy and public health interventions, and resulting in loss of life (Proctor, 2008). Even with more benign intentions, though, determining who and what to trust has become more challenging owing, in large part, to the rapidly growing scale of connectivity and the ways that information is packaged and presented through social media (Butcher, 2021; O’Connor & Weatherall, 2019; Wang et al., 2019). This has been aggravated by increasingly polarized and confrontational rhetoric and fractiousness that have directly attacked the usual reliable sources and, indeed, the basic institutions of civil society (Habermas, 2023; Olaniran & Williams, 2020). The situation prompts consideration from cultural psychiatry because it troubles our patients (and ourselves) and overlaps with basic questions of pluralism, relativism and epistemic justice. And, though, I hesitate to apply a psychiatric vocabulary to social problems (which usually amounts to little more than name calling), it does look like a form of collective pathology. What is going on and what can we do about it?

My title comes from the work of the mid-20th century scholar Alfred Korzybski, who saw in science and mathematics a way to improve common habits of thought and instill greater rationality into human thinking and decision making (Korzybski, 1958). He founded the discipline of General Semantics, which aimed to give an account of how we produce meaning that would have prescriptive value for civil society and human survival.^1^ One of his famous aphorisms is “the map is not the territory” (Korzybski, 1941). This helpful reminder encourages us to distinguish our models and metaphors from the world we traverse, but it was upended by Marshall McLuhan, and later constructivists, who insisted that the medium is the message (McLuhan, 1964). We now recognize that maps and metaphors create new (virtual) territories that we increasingly inhabit (Siegert, 2011). In this time of infoglut and polarization with internet bubbles and echo chambers, masking and doxing, the territories and topologies of media pose exceptional challenges to adaptation and survival. This encourages us to look at the ways in which knowledge and reasoning are embedded in social contexts.

Social epistemology draws attention to the social embedding and production of knowledge (Bueter, 2019; Fricker et al., 2020; Goldman, 1987; Goldman & Whitcomb, 2011).^2^ Knowing and believing are not simply individual cognitive processes but are based on participation in social systems and forms of life. As such, knowing is intertwined with social structure and process, which can be understood in terms of the dynamics of cultures and subcultures, networks and niches, styles of reasoning or habits of thought, and epistemic communities.

In this article, I want to consider problems of social epistemology that are central to our current predicament: How do we know what to believe? What are the sources of trust, epistemic authority, and styles of reasoning? How do we decide when and where to forage for information? How do we change our beliefs in response to new evidence, challenge or critique? How do we decide what can and cannot be known? What are the consequences for forms of social life and civil society of cultural pluralism that gives rise to different ways of knowing and warranting truth claims? Finally, how do these broad questions intersect with the concerns of cultural psychiatry?

## Rationality, irrationality and belief

Many psychiatric disorders involve pathologies of belief (Davies & Coltheart, 2000). We may believe things that are unfounded or distorted in ways that impede our functioning—as the cognitive theories of depression and anxiety suggest—or the underlying mechanisms of belief and conviction may be impaired in ways that make it hard for us to navigate the world, as seen in paranoia or delusional disorder (Connors & Halligan, 2017). The relationship between these forms of pathology and conspiracy theories—whether common or idiosyncratic and far-fetched—has been the focus of much debate (Bortolotti, 2024; Cassam, 2019; Douglas & Sutton, 2023; Galbraith, 2021; Gold & Gold, 2015). The ability to recognize real conspiracies may be adaptive and is certainly politically essential. Common ‘garden-variety’ conspiracy theories may simply reflect our constant search for patterns to explain troubling events, which is heightened in times of crisis and confusion. But pervasive conspiracy theories that undermine the person's self-confidence, trust in others, and social institutions are maladaptive for both individuals and societies, and may reflect cognitive, affective or social pathology.

Analytic philosophers have approached belief in terms of propositional attitudes; that is, as a matter of the qualification, stance or attitude that one takes toward certain statements or claims about oneself and the world (Kelly, 2002). The challenge then is characterizing precisely what this stance adds to the statement itself. If I say, “I believe the sun is shining,” how does that differ from saying simply, “The sun is shining”? The former seems to convey additional information about my position—depending on pragmatic context, emphasis or inflection, this might be a declaration of commitment or a qualification or even an equivocation. Pragmatically, this has to do not simply with questions of truth but with epistemic questions about the source and type of conviction associated with knowledge claims and experience.

There is evidence from cognitive and social psychology of the limits of this approach, which fails to capture the ways that beliefs actually function, including how they are formed, applied, and persist or change in the face of new information, counter-evidence, or social influence. Moreover, the forms of rationality assumed by these models do not capture much of the structure of everyday practical reasoning, which involves bounded or limited rationality, many types of cognitive bias, as well as strong dependence on context and emotion (Bortolotti, 2015, 2020; Gigerenzer, 2020; Henrich et al., 2001; Kahneman, 2011; Nilsson, 2014). Efforts to develop more accurate and inclusive accounts of the nature of reasoning lead to an emphasis on framing, context-sensitivity and an ecological view of knowledge and conviction as always socially situated and dependent on networks of meaning and practice (Fiedler & Wänke, 2009). This, in turn, requires rethinking our view of beliefs. Instead of being codified in explicit propositions, many or most beliefs involve implicit assumptions about how the world works, engagements with particular epistemic practices, commitments to social relationships and institutions, tacit background knowledge, and cultural ontologies. Because of their embodiment and enactment as epistemic practices, and their embedding in social relationships and institutions, direct challenges to beliefs or counter-evidence are often met with various forms of resistance or rejection (Festinger, 1956; Kirmayer, 1990; Klintman, 2022).

Traces of the view of belief as propositional knowledge and attitudes can be found in anthropology. Allan Young (1981, 1982) critiqued the implicit rationality of the explanatory model perspective in medical anthropology, which assumed that people could give reasoned and coherent accounts of illness causes, mechanisms and outcomes, when, in fact, people often reason analogically from prototypes or work metonymically with the tacit structures of everyday experience. Byron Good (1993) described the implicit rationality of biomedicine itself, noting the contrast between medicine, which claims a basis in scientific facts, and patients’ culturally based interpretations, which are termed “beliefs”. The assumption is that “we” (as practitioners of a rational scientific medicine) have knowledge, whereas “they” (as participants in a cultural system with a folk ontology) merely have beliefs. While conveying the facts of biomedicine, this asymmetry leads to discounting and discrediting other knowledges or merely tolerating others’ practices as the more or less unfortunate consequence of their (ill-founded, at times even unintelligible) convictions. A growing literature on epistemic injustice documents the problems in clinical communication and ethical practice that arise from this assumption (Crichton et al., 2017). In the case of psychiatry, where much of our theory is grounded in cultural concepts of the person that vary across cultures, the one-sided promotion of mental health literacy can contribute to epistemic injustice as part of a broader project of cultural proselytization.

The recognition that cultures include systems of knowledge and practice that may be grounded in alternative ontologies has led to recent challenges to the dominance of scientific rationality as the paradigm within which diversity must be understood. Challenges to the dominant episteme come from those concerned to redress the colonial suppression and annihilation of alternate worldviews, ways of knowing, and systems of values (Aikenhead & Ogawa, 2007; R’boul, 2022). Alternative frameworks come from intercultural philosophy, postcolonial studies, and other disciplines that challenge the dominance of science, which is usually framed as a western institution (Elshakry, 2010). Yet there is much in science (as an institution and form of social practice) that seems to offer us unique values, virtues, tools and protocols of wide applicability. How can we reconcile cultural and epistemic diversity with the need to have a firm empirical grounding in many domains of activity, a rational deliberative process to resolve disagreements, and maintain a cohesive and inclusive pluralistic civil society? This challenge is especially urgent when faced with wholesale rejection of scientifically based public health measures or widespread beliefs in outlandish conspiracy theories.

## An ecosocial view of knowledge and knowing

Generally, a bare concept of norms abstracted from their social context is not sufficient to judge whether knowledge claims or beliefs are well-founded, dubious or pathological. To get a better picture of the origins and grounding of everyday beliefs, misinformation, or engagement with conspiracy theories, as well as pathological phenomena like delusions, it is useful to make use of notions of embodiment, enactment, embedding and extension that are core concepts of 4E cognitive science (Kirmayer & Ramstead, 2017). Here, embodiment stands for the ways that the body (our anatomy and physiology and the resultant phenomenology) shapes experience—as well as the way our engagements with the world reshape our bodies. As a result of processes of embodiment, everyday knowledge and conviction are often conveyed through bodily felt experiences that seem unmediated. Their very immediacy warrants their veridicality. The concept of enactment emphasizes that our bodily experience is not simply a process of building up a picture of the world, but one of active engagement with the world in ways that aim to maintain our functioning and achieve our goals. Cognition and perception serve action through ongoing sensorimotor cycles that include brain, body and world. What is experienced as unmediated, in fact, arises from these cycles.

The processes of embodiment and enactment then are embedded and extended; that is, they always occur in a nested series of contexts: the body itself and its interoceptive processes and possibilities for sensory orientation and movement; our immediate environment and relationships with others; and the larger social networks and niches through which we collaborate with others. The human environmental niche is fundamentally social: co-constructed through interactions with others. The social environment provides a wide array of cultural affordances, potential modes of perception and action that depend on understand the cultural meanings and scripts (Ramstead et al., 2016). We learn how to perceive and act with cultural affordances through developmental processes that involve specific regimes of attention. In effect, we learn to think with and through other minds, whether present as people in our local world, or accessed through the structures and traces other people leave in our institutions and landscape (Veissière et al., 2020).

Accounting for the cultural shaping of experience then requires that we move beyond the individual-centric view that dominates psychology and psychiatry. Culture is not entirely a matter of personal agency—indeed, it is mostly not a matter of the agency of the person at hand but of others—historical forebears, family, clan, community, institutional authorities—all manner of others who frame one's life and to whom one must answer. Of course, those others are constrained, in turn, by a social system with its history, exigencies, imperatives and momentum. Understanding how this larger landscape of meaning interacts with our efforts to make sense of the world is a central concern for cultural psychiatry and points toward a situated view of mind, brain and person.

## Conviction, active inference and the Bayesian brain

Current views of the brain emphasize that its primary and most general function is to maintain the life and viability of the individual by predicting changes in the environment (Clark, 2015). The same methods of Bayesian prediction that govern basic cognition may also account for other aspects of self-regulation and allostasis (Pezzulo et al., 2015). The notion of predictive processing has been elaborated in models of active inference, in which the aim is not only to update models to more accurately predict the environment, but to act on our body and the world to make them more closely conform to our needs and goals. The active inference process is driven by discrepancies between prediction and observation that lead to updating of predictions, corrective actions to make the body or world confirm to expectations, and information foraging to identify resources that allow better prediction. Crucially, in active inference, sources of information about the world are also associated with weightings of their precision or reliability (Parr et al., 2022). In computational models of cognition, changes in the weighting of expectations or of external sources of information can lead to forms of fixed belief analogous to delusions (Adams et al., 2022).

Although framed as a theory of individual organismic survival and adaption, the boundaries of the cognizing agent in active inference are flexible. In modeling, these boundaries are formalized as Markov blankets, boundaries across which one has only limited statistical information about events from which the actual (hidden) states of the world must be inferred. The Markov blankets of human cognition can be flexibly extended to include our bodily extensions (glasses, gains, books, computers) and interaction with others (Clark, 2017; Kirchhoff & Kiverstein, 2019). In particular, humans are constituted as knowers in relationship to each other, co-generating and sharing experiences, facts, hypotheses, and conjectures about the world. This exchange occurs not through an even-handed sharing of all information with everyone, but within specific social institutions, communities, niches or networks. In some cases, these networks may constitute epistemic communities—defined and delimited by commitments to specific ways of knowing (Knorr-Cetina, 2007). These communities may form in response to shared cultures, identities, and interests and also in response or resistance to dominant epistemes (McHugh, 2017). Characterizing the dynamics involved in the production and circulation of knowledge involves understanding the networks that connect (and isolate people) and the epistemic spaces or niches they occupy (Navin, 2013).^3^

**Figure 1.**
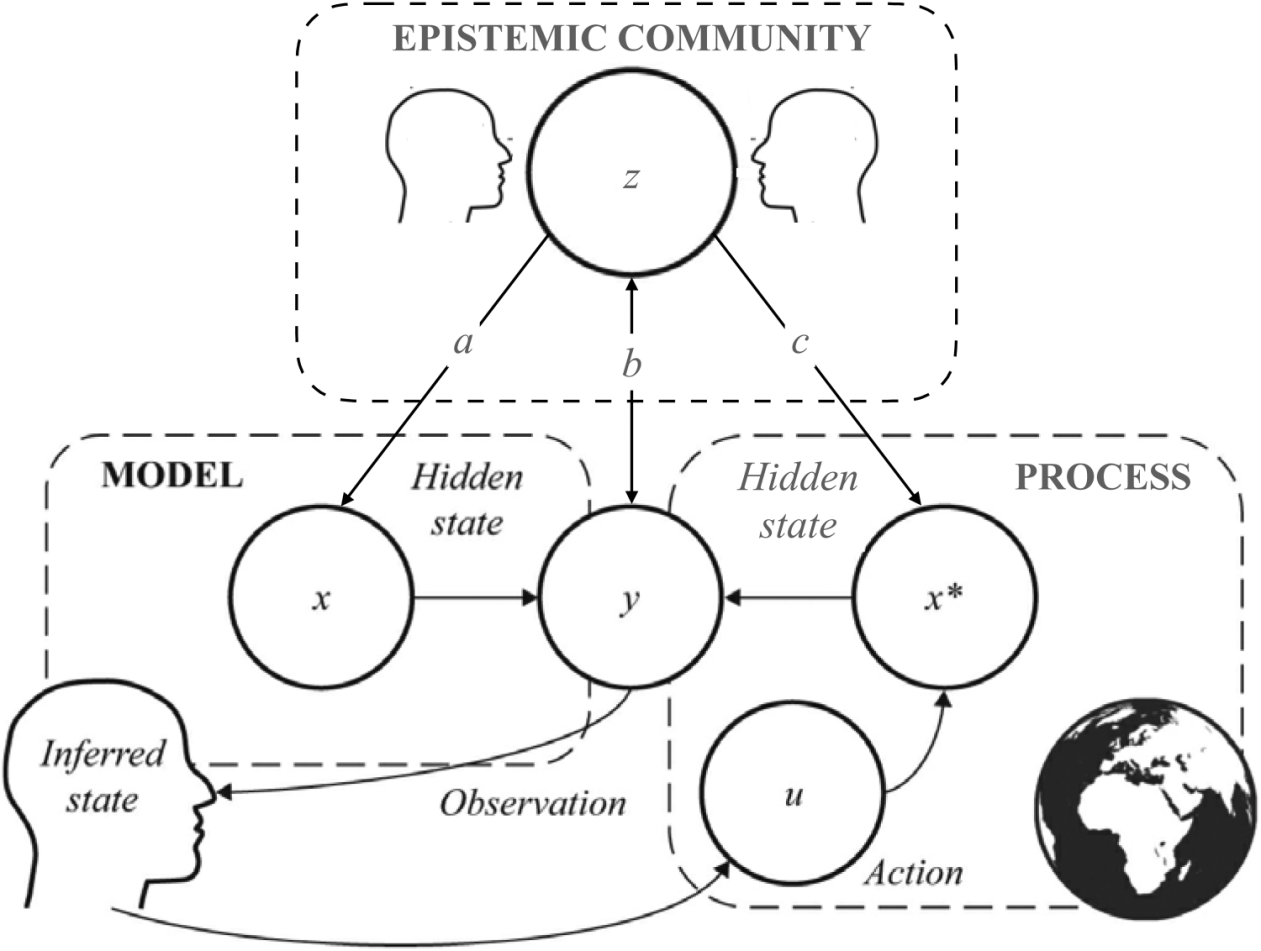
Active inference and the influence of epistemic communities. The lower part of the figure (adapted from Parr et al., 2022, p. 24, MIT Press, CC) represents the process by which individuals infer the state of the world through active inference. This figure adds another layer labeled “epistemic community” that consists of groups of interacting individuals engaged in active inference under specific regimes of attention. Epistemic communities influence this process in three ways: (a) installing priors about the world (e.g., as a dangerous place, full of threats, untrustworthy leaders, and unreliable sources of information); (b) shaping the process of observation of the world (e.g., through media exposure); and (c) creating events and processes in the world (e.g., real conspiracies or insurrections).

The active inference framework introduced above can be extended to include the dynamics of epistemic communities (Figure 1). These communities are composed of individual knowers who operate with the same principles and who are coupled with each other in ways that result in strong mutual influence. This can lead to local consensus but also, in some instances, to increasing polarization of views within and between communities.

Epistemic communities (as a group of active inference agents united by shared priors and objects of attention or concern) can influence an active inference agent extrinsic to their group in three main ways: (a) by installing new priors that influence the expectations and information foraging of the individuals; (b) by influencing the channels of communication through which the individual observes the world; and (c) by acting on the world itself (in both direct and indirect ways, that is, for example, by influencing others) to change the underlying processes. This very general framework provides ways to approach our current predicament and clarify what is new and challenging in contemporary ecologies of information. In particular, the dynamics of epistemic bubbles (in which we are exposed to the views of only those who are like-minded) and echo chambers (in which we discount the epistemic authority of those who do not share our identity or allegiances) (Nguyen, 2020)—both of which may reflect our own agency as well as the effects of social media platform algorithms and manipulations—can be modeled as features of these ecologies (Albarracin et al., 2022).

The priors that epistemic communities install may concern purported facts about the world (the election was stolen), but also ontologies (there are hidden cabals that control the world); and epistemic assumptions about the availability and reliability of specific sources of information in general or for specific domains (e.g., reliable information on climate change is provided by scientists, religious leaders, the leader of my political party, or rich businessmen). Observations of the world depend on background knowledge, position, and perspective, but also on access to information, which is mediated by our social network, access to experts, and social institutions. This is increasingly dominated by social media. Finally, epistemic communities can work behind the scenes to reconfigure the world to change the landscape within which we forage for information. This can occur by changing the dominant theories and metaphors that influence the reception of information or by changing the architecture and algorithmic regulation of inter-community networks and communication. This may hive us off in separate communities or distort the representativeness of the environments we are able to explore (epistemic gerrymandering). Unfortunately, the resultant landscape can be much smaller than or disconnected from the larger contexts that actually determine our fate, as individuals and as a species.

## Social epistemology and the pandemic's “new normal”

The prolonged uncertainty, anxiety, and restrictive public health measures associated with COVID were described as “the new normal” (Corpuz, 2021). “New” carries the connotations of novelty, singularity, and uniqueness, whereas “normal” here stands for normalization, a response to the persistent situation of crisis, which hides the social, cultural, and political production of everydayness (Sunstein, 2021). This new normal confronted us with a set of problems in social epistemology.

A social perspective on epistemology begins with the recognition that knowing and believing are not simply individual cognitive process but fundamentally based on participation in forms of life. As such, knowing is intertwined with social structure and process, which can be understood in terms of culture, social niche, habits, and community. This includes features that are presented to us as cultural affordances—potential modes of our local niches or larger social contexts that depend on cultural practices. The social dimensions of epistemology also concern the politics of knowledge as power (Foucault, 2007; Han, 2018). Both the local and distal ecologies of knowledge and the dynamics of power contribute to how and what we know or can know.

The pandemic threw into high relief a set of longstanding questions about epistemic authority associated with modernity, secularization, and industrialization. Epistemic authority is used to warrant attention to and action on social problems—amplifying the sense of danger, risk, vulnerability and urgency, and the potential for corrective action, which then drive epistemic authority. The displacement of religion and its spiritual and institutional authority by science and technology, characteristic of modernity, has been a partial process that has worked well only for a segment of the population. Science education in the general population is limited and the inevitable uncertainties in research findings undermine confidence in its authority among those who do not grasp its epistemology (Covitt & Anderson, 2022). For this and other reasons having to do with identity and community, many people turn to other sources of authority to resolve uncertainty in decision making about major life events. The pandemic had features that presented specific epistemic conundrums: large scale (the pan in pandemic); massive uncertainty, inconsistency, contradiction, and confusion; the illusion of a simple causal agent (the virus), which implied there ought to be simple explanations of causality and corresponding attributions of responsibility and blame, as well as simple solutions; the rapid spread of (dis)information through the internet and other media in what was termed an “infodemic” (The Lancet Infectious Diseases, 2020; Zielinski, 2021).^4^

In the case of the pandemic, settling on basic “facts” including the scope of the pandemic, its origins, and appropriate prevention or treatment on which to base action posed many challenges, reflecting the pace and methodological limitations of scientific research. Questions persisted about the scale, magnitude, incidence, rate of spread of the infection, the origins of the virus, and effective methods of prevention and treatment. There was great uncertainty about the number of cases: the reporting of incidence rates was technically challenging, and there was deliberate hiding, minimizing or manipulation of those figures for political reasons (Billig & Marinho, 2023). Counting depends on diagnosis, which involves case finding and accurate testing; asymptomatic cases were common; estimates of surplus deaths could include indirect effects of both pandemic and response, including mitigation efforts (reluctance to go to hospital, impacts of social isolation, etc.) (Ibrahim, 2020; Manski & Molinari, 2021; Russell et al., 2020).

Origin stories are the fount of myth making and the COVID-19 pandemic was no exception. Claims about the origins of the virus were driven by political interests and concerns—and deliberate attempts to assign or deflect blame. Genetic epidemiological strategies for establish origin take time and data. Conspiracy theories were rife (bat caves, pangolins, animal markets, research labs, bioweapons) (Birchall & Knight, 2023; Freeman et al., 2022; Nattrass, 2023; Stein et al., 2021; Sturm & Albrecht, 2021). Although some of these conspiracy theories had nuggets of truth, many were far-fetched. And even if some potentially relevant elements were identified, an adequate account would have to consider the interaction of multiple factors rather than the single “big” revelation that captures public attention.

Public health efforts to slow the spread of infection depend on probabilistic reasoning and reducing the risk of an event that may not happen. The beneficial effects of social distancing, use of protective equipment, limitations on mobility and other measures were delayed and general, whereas the inconvenience, loss and harms of these interventions for the general public were immediate and specific. Opposition to masks and social distancing was based partly on concern about the negative effects of these preventive measures, partly on mistrust of authorities (again framed not so much against professions but against what was perceived or portrayed as partisan politics), and partly on the proliferation of internet misinformation (Caceres et al., 2022; Gabarron et al., 2021; Tasnim et al., 2020). We have basic emotional systems of evaluation and response related to infection that play a role in pandemic response (the laws of contagion, the mechanisms of disgust, purity and danger) (cf. Curtis, 2013; Curtis & de Barra, 2018), and ordinarily these would be sufficient to motivate preventive measures. But, for many, overarching social and political concerns trumped more basic threat perception and response.^5^

In the case of vaccine resistance, anxieties about biological interventions and increasingly elaborate conspiracy theories played a role for many people (Birchall & Knight, 2023). Most medical interventions are directly beneficial to individuals. In the case of public health prevention measures, the benefits are mainly to others. Public health directives on masking, social distancing and the use of public spaces were viewed through the lens of libertarian civil society and the commons, and denounced as infringements on freedom and liberty (Sunstein, 2021). In the US and some other neoliberal regimes, the notion of the common good seemed to have worn thin or to have been deliberately undermined by right-wing ideologues.

The COVID pandemic saw many claims of efficacy for untried, implausible or obviously dangerous treatments (e.g., hydroxychloroquine, ivermectin, bleach!) (Ghai et al., 2024; Perlis et al., 2023; Pilkington, 2020). Determining effective treatments for specific types of disease poses its own distinct epistemic challenges. When rates of infection or recovery are not well known, establishing the benefit of interventions in observational studies is limited by the absence of comparisons or controls. In open trials, placebo effects may shroud the ability to detect specific efficacy. Ultimately, determining efficacy requires clinical trials, which take time and large numbers. Despite the lack of such information—and, eventually, clear indication of lack of efficacy—many people endorsed and tried these treatments based on advocacy by politically motivated endorsements and presentations on social media. In practice, of course, people often follow advice of friends and neighbors either from conviction or a “why not” strategy—which may be benign unless it leads them to reject effective treatments or suffer from serious side effects from unproven interventions. In this case, the severity of the potential illness, the endorsement by highly partisan media figures, and wishful thinking resulted in continued interest in dubious or ineffective treatments despite the weight of scientific evidence and professional opinion against them.

Although the COVID pandemic highlighted problems in the social production and dissemination of knowledge, we face similar epistemic dilemmas in many other arenas, including the proliferation of disinformation and deliberate “fake news” (especially by those who accuse others of it) (J. Rose, 2020). In the US, this has included disinformation campaigns related to the global climate crisis, advocacy for gun rights as a pillar of collective identity despite the devastating effects of gun violence, increasing right-wing and white supremacist radicalization, and systematic attempts to disqualify political opponents, undermine the democratic political process and sabotage the peaceful transfer of power (Galison & Proctor, 2020; Michaels, 2006; Oreskes & Conway, 2008). Though each has a distinct history and trajectory, these arenas or campaigns of ignorance and disinformation share some common features reflecting the impact of the internet and social media in increasing the speed, breadth and reach of particular voices and positions from the margins (L. Rose & Bartoli, 2020) and the rise of populist politics and the erosion of the values of liberalism and inclusion (Habermas, 2023). All of this is driven by the structural violence and social inequities that amplify economic disparities (Milanovic, 2016) and occurs against the backdrop of the global climate crisis that feeds a general sense of impending catastrophe (Bould, 2021).

The scale and scope of these changes and the corrosive effects on civil society point to the urgency to think through the intersection of politics, information ecologies, and epistemic values in multiple domains. It useful to ask what is common or unique to the different issues around which we see epistemic conflict and polarization, including climate change, gun violence, and political polarization itself.

## Ecologies of information: grounding knowledge in empiricism, community, and critique

The spread of information and the ways it is taken up by different individuals or groups can be tracked as a kind of epidemiology of representations (Sperber, 1985). The analogy with epidemiology and contagion is inexact, however, because individuals are active information foragers and ideas are not discrete, fungible units of information or factoids, but generative images and metaphors that are linked to larger narratives that are part of particular ideologies and institutions. In effect, information comes prepackaged with interpretations, framed in particular ways that make them emotionally charged, and associated with specific identities, affiliations, and social positions. Certain kinds of stories are especially appealing and more likely to “go viral” because they tap into common cognitive biases (Boudry et al., 2015), as well as pre-existing prejudice or ideologies (Cassam, 2019) and hence mobilize strong affect, identification, and social positioning.

This is well illustrated by the long tradition of paranoid thinking in US politics (Hofstadter, 1965). As a cognitive and interpretive style, this paranoia is based on the conviction that there are hidden, malevolent forces at work in the world and the realization that, in novelist Thomas Pynchon's words, “everything is connected” (Pynchon, 1973) through “other orders behind the visible” (Bersani, 1989)—indeed, “God is the original conspiracy theory” (Sanders, 1975). In recent years, a particular version of this conspiracy-oriented worldview has become increasingly popular and mainstream, and has been largely adopted as a style (and even an explicit platform) by the Republican Party (Appelrouth, 2017). The irony is that those who like to call themselves “conservative” increasingly adopt attitudes to truth and public debate that are radical and highly corrosive to civil society and antithetical to conservative values and institutions.^6^ This does not mean, I should add, that those who subscribe to such conspiracy theories are clinically paranoid.^7^ The pathology here is social not psychiatric.

All systems of belief (or sporadic beliefs) invoke authority structures. Epistemic authority itself may be warranted by different means from the knowledge it warrants (e.g., group affiliations, religious commitments, ethnic identities). This might lead us to adopt styles of reasoning that are based on the ex-cathedra statements of an authority figure but, more interestingly, it could lead us to ground our convictions in appeals to bodily feelings or intuitions that are, in fact, the outcome of implicitly installed biases of attention and evaluation. So, we trust CNN, MSNBC or Fox News according to our tribal allegiances (which we may experience as aesthetic preferences), but then receive a barrage of images and stories that reorganize our expectations and information foraging in the virtual worlds of screens and beyond. The dilemma is that, although we may be aware of our initial choice of allegiance (and able to give a reasoned account of why we chose it), the consequences of this choice are largely implicit and outside conscious control. This points to the need for critical analysis of the dynamics of media exposure and efforts to uncover the design of the algorithms that capture and canalize our attention (Hari, 2022; O’Neil, 2016).

We are living in local and global information ecologies that have toxic effects for individuals, communities, and global civil society. This involves both intentional exploitation of misinformation and deliberate production of disinformation (Adler & Drieschova, 2021; Cosentino, 2020), as well as unintended and unanticipated effects of connectivity through the vehicles of social media. The media platforms themselves continue to grow, with governmental limited regulation, despite their harmful effects, because they provide enormous economic gain for corporations and individuals.

In our current information ecology, the sources of information are proliferating while trust in traditional sources is fast eroding—through direct challenges and efforts at disqualification as well as deliberate exploitation of the dynamics of information foraging. Responding to this epistemic challenge requires rethinking the social basis or facilitators of empiricism, rationality, deliberation, and decision making both in terms of adaptive value and contribution to forms of life that we value ethically and aesthetically.

In this context, it seems important to revisit the claims for science as a social institution and practice that has something vital to offer civil society beyond advances in technology. This requires characterizing what is specific to science as a social epistemic practice (Pigliucci, 2015; Pigliucci & Boudry, 2013). As Harry Collins and colleagues argue, science has some specific values and virtues that recommend it as a crucial element in any form of life we imagine might speak to our current predicament (Collins, 2023; Collins & Evans, 2017; Collins et al., 2020; Collins & Pinch, 1998, 2008). The epistemic virtues of science include: recognition of the unfinished, incomplete, and tentative nature of knowledge that leaves room to discover new facts, models, and metaphors and better understand their implications; tolerance of uncertainty, avoiding premature foreclosure; an intolerance of ambiguity, pushing for clarification so that competing views can be compared and tested; a willingness to adhere to rigid rules of argument, demonstration, and accountability, so that it is possible to learn from unexpected consequences; an openness to critique through reasoned argument and counter-evidence; and a commitment to conservatism, keeping what works and requiring that it be subsumed by new more accurate or encompassing frameworks.^8^

None of these values are unique to science but taken together they mark off a particular form of life and intentional community that offers a contrast with the narrow interest driven activities of partisan politics, marketing and religious indoctrination. These virtues are too important to be discounted as simply reflecting the interests of a particular cultural or historical group (Collins & Evans, 2017).

## Pluralism and the ethics of knowing

The trustworthiness and objectivity of science results from specific kinds of social process that include openness, transparency, and commitment to truth (Collins, 2024; Oreskes, 2019). The interplay of diverse voices and critical perspectives is an essential part of this process. Oreskes argues that “objectivity is maximized… when the community is sufficiently diverse that a broad range of views can be developed, heard, and appropriately considered” (2019, p. 6). But when sources of epistemic authority differ and make claims that are in direct conflict, we may face serious challenges in finding common ground.

Cultural communities may build their collective identity around particular bodies of knowledge and ways of knowing that seem incommensurable with the frameworks of biomedicine. The dilemma for intercultural work is that in the case of epistemic communities, knowledge claims are not simply statements of (believed in) fact, but simultaneously expressions (affirmations) of identity and commitments to a way of life or survivance of a community (Kirmayer, 2012). This means that challenges to the credibility of knowledge become challenges to the legitimacy or viability of the community. Further, for those who see epistemic commitments as central to identity, the knowledge claims of others are prima facie evidence of their own identities and allegiances. This undercuts the possibility of a shared understanding of “the facts” and hence of the potential to resolve conflicts about matters of fact as a prerequisite to debate about the implications of alternative courses of action, which can be weighted in terms of hierarchies of values and desired outcomes.

Current efforts to respect Indigenous knowledges in healthcare research and clinical decision making provide an arena in which some of the challenges of reconciling alternative epistemologies can be clearly seen (Cohen-Fournier et al., 2021). Responding to the history of marginalization and oppression, Indigenous scholars have advocated for the value of Indigenous knowledges and ways of knowing. Sometimes these knowledges are described as fundamentally empirical; that is, based on practical knowledge, observation, and experiment to find what works. In other instances, knowledge claims are based on appeals to the depth of oral tradition and practices, traced back to creation stories, or connected to a sacred relationship with the land, nonhuman others or the creator. These forms of warranting truth are based on particular ontologies, identities, and commitments to communities, or ways of life that may not be shared with or even available to others. Though originally rooted in a particular culture and place, these knowledges have been put into wider circulation through exchanges between communities and, increasingly, through the internet. To some extent, this circulation de-territorializes this knowledge, detaching it from its specific connections to land and place and encouraging others to find parallels with their own life situations.

Whatever their warrants, however, these bodies of knowledge cannot address all of the concerns raised by a health problem (like COVID) or an anthropogenic catastrophe (like climate change) for several reasons: (a) they are originally based on small-scale societies and local contexts that may not be generalizable to other scales and contexts; (b) they may be valued not for their practical efficacy, but for the ways they affirm identities and worldviews that have intrinsic value for the culture and community; (c) they may be consistent with a relational ethos that is a good foundation for a community but that does not explicitly address the problem of cultural diversity in the larger society. Blanket acceptance of diverse epistemologies can not go very far in resolving the conflicts that arise in healthcare and politics where too much is at stake.

Part of the problem may be the way we use current models of rationality and decision making to understand popular beliefs and practices. The analysis of rationality is a blunt instrument for distinguishing problematic healthcare decisions. There are issues of the moral weighing of person versus public risk that also need to figure in our evaluation of others’ positions. Whatever their logic, the errant decision making on display in responses to the pandemic and in current political polarization seems to reflect ideological commitments that conflate political power with epistemic authority. In the case of the US, there may be an underlying faith in the “marketplace of ideas” as a path to truth. But as political philosopher Wendy Brown suggests, “Both Nietzsche and Weber would groan at the marketplace of ideas often hailed as truth's determinant today, as if markets and truth were ever related, as if markets secure and refine rather than abuse and degrade truth, and as if targeting and trolling had not become permanent features of all marketplaces, including those of ideas” (Brown, 2023, p. 30–31). The same distortions plague public attitudes toward science in healthcare and encourage us to be skeptical about claims for authoritative knowledge that are driven by commercial interests or ideologies.

Similar skepticism should apply to claims that certain practices (or medicines) are well-founded because they have stood the test of time—as if time itself is the arbiter of truth. In the case of cultural traditions or practices this amounts to a forme fruste of social Darwinism: survival of the practices that are the fittest. But we do well to remember that Darwinian fitness is reproductive fitness; on this analogy practices that contribute directly or indirectly to reproductive fitness may persist and, if replicable, prevail over time. This means that practices that contribute to other distal or disparate goals or values may not be selected for. Even when there is a net gain in reproductive fitness, it does not mean the benefits are evenly distributed. In fact, practices persist for many reasons including their fit with social structures and systems of power that are resistant to change. It is a commonplace, that practices that are harmful to some or many persist for long periods, provided only that they benefit those in power. The duration of persistence reflects the speed with which the negative effects spread and how decisive they are for the population. Using a hair pomade that causes cancer might be popular for a long time if it confers aesthetic advantages in sexual selection, and binding feet in ways that impede mobility may work for a privileged class able to be carried about. So the claim that “this medicine must work because it has been used for millennia” is compelling only if there have been explicit empirical tests (proto-science), alternatives are available and have been compared and found wanting, and there is an unimpeded path from evidence of effectiveness to the decision to use the medicine. In reality, competing values and concerns other than therapeutic efficacy may contribute to the persistence of healing practices. These other uses of knowledge must be considered to make sense of the value and limitations of epistemic pluralism. Many competing knowledge claims can be resolved by recognizing that they have different domains of application or goals. What at first appears incommensurable may then then become complementary.

## Conclusion

Science can provide a partial antidote to the toxic brew of lies, misdirection, and conspiracy theories that impede effective public health and pollute political rhetoric. The problem we face is not with deficient rationality per se, but with a lack of background knowledge and erosion of trust in institutions that can provide reliable information. These are consequences of education and civic engagement, but also of the overarching ecology of knowledge and information in which we are embedded. Rather than focusing on individuals’ irrationality, then the “solution lies with managing the epistemic environment” (Levy, 2023, p. 944). Thinking in terms of ecologies of knowledge may allow us to work out the dynamics of epistemic communities both in their internal consequences for the decision making of participants and in their externalities, which manifest as forms of civil society that can allow equity, diversity, and deliberative democracy.

The analysis of epistemic communities suggests three broad approaches oriented toward specific targets: (a) altering the priors that govern individual cognition, action, and perception; (b) regulating the channels through which we access and receive information about the world; and (c) working toward social change that reduces structural violence and repairs and protects the forms of social life that enable coexistence.

The ways we perceive the world depend on our knowledge and expectations or cognitive priors and these result from life experience including education and participation in public debate. This points to the need to improve education about the epistemic virtues of science as an institution committed to systematic inquiry, truthfulness, transparency, accountability, tolerance of uncertainty, recognition of cognitive and social biases, and evidence-based reasoned critique. Education about other institutions (government, law, healthcare) can also foster the trust in institutions in ways that foster civil society and deliberative democracy.

Social media have powerful effects on how we access information about the world. Far from offering broad vistas, they have become narrow portals manipulated by political and commercial interests, using algorithms that seek to monetize human attention by capitalizing on our emotional and epistemic vulnerabilities. Regulating social media and the internet is thus an urgent priority. This regulation must allow us to uncover hidden interests and interdict (and prosecute) malign actors. Such regulation requires state interventions; it cannot be left to corporations that are in fundamental conflicts of interest. In addition to curtailing these deliberate attacks on civil society and individual well-being, we need to clarify the inadvertent consequences of the scale and speed of the internet and counteract the amplification of misinformation and promotion of disinformation that contribute to harmful polarization.

Strengthening civil society will also require direct action to counter deliberate efforts to discredit and destroy democratic institutions. This includes uncovering and dismantling real conspiracies (like election interference by foreign governments and their proxies; see: Henschke et al., 2020) and taking legal action against antidemocratic actors. Tracing disaffection and ressentiment to its roots, we need to address the growing inequalities and inequities in local and global society that lead to marginalization and exclusion. Most basically, we need to create spaces of inclusion in which we can engage in the kinds of open dialogue that promote mutual recognition and respect for the other.

## Notes

1. Korzybski (1958) argued for the importance of “non-Aristotelian” logics in which the law of the excluded middle does not hold; that is, logics in which truth is multivalued, multidimensional, probabilistic or fuzzy. A variety of such systems have been developed, including fuzzy set theory with relevance to reasoning about natural categories (Sadegh-Zadeh, 2000). These logics take on new significance in the light of discussions of alternate epistemologies (Reiter, 2020) and efforts to develop quantum–theory-like models of cognition (Pothos & Busmeyer, 2022).

2. There are several distinct approaches under the broad banner of social epistemology that vary in their views of the ability of science or other social institutions to produce “truth” (Niiniluoto, 2020).

3. The notion of epistemic communities then needs to consider history, geography and above all, politics. In the wired world of social media, epistemic communities do not respect national boundaries, but follow the lines of social media consumption. For example, the uptake of conspiracy theories in Canada reflects consumption of online material from US X (formerly Twitter) accounts (Bridgman et al., 2022).

4. For a critique of the infodemic metaphor see: Simon and Camargo (2023). The spread of misinformation reflects both social and psychological dynamics that differ in many ways from the spread of an infectious disease (Greenspan & Loftus, 2021; La Rocca et al., 2023).

5. Disgust can amplify these social and political dynamics as well (Kam, 2019).

6. On rescuing the word “conservative” for its rightful use meaning to preserve, protect, and uphold what needs to be (ought to be) conserved of traditions over time, see “Rescuing conservatism: A defense of existing value” in Cohen, 2012, pp. 143–174). Of course, this conservatism requires constant adaptation to changing contexts so that what is preserved is value not empty form.

7. Indeed, Alsuhibani et al. (2022) describe differences in some of the personality traits and characteristics associated with paranoia and those associated with the tendency to endorse conspiracy theories. Then too, narcissistic personality traits may lead individuals to see the world mainly in terms of its ability shore up their fragile self-esteem resulting in a style of thought that sociologist Fred Goldner called “pronoia” (Kirmayer, 1983).

8. For discussions of the virtues of science from social–historical, epistemic, political, and pragmatic perspectives see: Collins, 2023; Gellner, 2013; Lapsley & Chaloner, 2020: Laudan, 2012; Oreskes, 2019; Van Dongen & Paul, 2017.

## References

[bibr1-13634615241296301] Adams R. A. Vincent P. Benrimoh D. Friston K. J. Parr T. (2022). Everything is connected: Inference and attractors in delusions. Schizophrenia Research, 245, 5–22. 10.1016/j.schres.2021.07.032 34384664 PMC9241990

[bibr2-13634615241296301] Adler E. Drieschova A. (2021). The epistemological challenge of truth subversion to the liberal international order. International Organization, 75(2), 359–386. 10.1017/S0020818320000533

[bibr3-13634615241296301] Aikenhead G. S. Ogawa M. (2007). Indigenous knowledge and science revisited. Cultural Studies of Science Education, 2(3), 539–620. 10.1007/s11422-007-9067-8

[bibr4-13634615241296301] Albarracin M. Demekas D. Ramstead M. J. Heins C. (2022). Epistemic communities under active inference. Entropy, 24(4), 476. 10.3390/e24040476 35455140 PMC9027706

[bibr5-13634615241296301] Alsuhibani A. Shevlin M. Freeman D. Sheaves B. Bentall R. P. (2022). Why conspiracy theorists are not always paranoid: Conspiracy theories and paranoia form separate factors with distinct psychological predictors. PloS One, 17(4), e0259053. 10.1371/journal.pone.0259053 PMC8989304 35389988

[bibr6-13634615241296301] Altay S. Berriche M. Acerbi A. (2023). Misinformation on misinformation: Conceptual and methodological challenges. Social Media + Society, 9(1), 1–13. 20563051221150412. 10.1177/20563051221150412

[bibr7-13634615241296301] Appelrouth S. (2017). The paranoid style revisited: Pseudo-conservatism in the 21st century. Journal of Historical Sociology, 30(2), 342–368. 10.1111/johs.12095

[bibr8-13634615241296301] Bersani L. (1989). Pynchon, paranoia, and literature. Representations, 25, 99–118. 10.2307/2928469

[bibr9-13634615241296301] Billig M. Marinho C. (2023). Preventing the political manipulation of COVID-19 statistics: The importance of going beyond diplomatic language. Language in Society, 52(5), 733–755. 10.1017/S0047404522000367

[bibr10-13634615241296301] Birchall C. Knight P. (2023). Conspiracy Theories in the Time of COVID-19. Taylor & Francis.

[bibr11-13634615241296301] Bortolotti K. (2015). Irrationality. Polity.

[bibr12-13634615241296301] Bortolotti L. (2020). The epistemic innocence of irrational beliefs. Oxford University Press.

[bibr13-13634615241296301] Bortolotti L. (2024). Is it pathological to believe conspiracy theories? Transcultural Psychiatry, 56(4). https://doi.org/10.1177/13634615231187243 10.1177/13634615231187243 PMC11629588 37525627

[bibr14-13634615241296301] Boudry M. Blancke S. Pigliucci M. (2015). What makes weird beliefs thrive? The epidemiology of pseudoscience. Philosophical Psychology, 28(8), 1177–1198. 10.1080/09515089.2014.971946

[bibr15-13634615241296301] Bould M. (2021). The Anthropocene Unconscious: Climate Catastrophe Culture. Verso Books.

[bibr16-13634615241296301] Bridgman A. Lavigne M. Baker M. Bergeron T. Bohonos D. Burton A. Loewen P. J. (2022). *Mis- and disinformation during the 2021 Canadian Federal Election* . Media Ecosystem Observatory, Centre for Media, Technology and Democracy, McGill University.

[bibr17-13634615241296301] Brown W. (2023). Nihilistic times: thinking with Max Weber. Harvard University Press.

[bibr18-13634615241296301] Bueter A. (2019). Social epistemology and psychiatry. In Bluhm R. Tekin S. (Eds.), Bloomsbury companion to philosophy of psychiatry (pp. 485–503). Bloomsbury.

[bibr19-13634615241296301] Butcher P. (2021). COVID-19 as a turning point in the fight against disinformation. Nature Electronics, 4(1), 7–9. 10.1038/s41928-020-00532-2

[bibr20-13634615241296301] Caceres M. M. F. Sosa J. P. Lawrence J. A. Sestacovschi C. Tidd-Johnson A. Rasool M. H. U. Fernandez J. P. (2022). The impact of misinformation on the COVID-19 pandemic. AIMS Public Health, 9(2), 262. 10.3934/publichealth.2022018 35634019 PMC9114791

[bibr21-13634615241296301] Cassam Q. (2019). Conspiracy theories. John Wiley & Sons.

[bibr22-13634615241296301] Clark A. (2015). Surfing uncertainty: Prediction, action, and the embodied mind. Oxford University Press.

[bibr23-13634615241296301] Clark A. (2017). How to knit your own Markov blanket: Resisting the second law with metamorphic minds. In Metzinger T. Weise W. (Eds.), Philosophy and predictive processing (Vol. 3, pp. 1–19). MIND Group. 10.15502/9783958573031

[bibr24-13634615241296301] Cohen-Fournier S. M. Brass G. Kirmayer L. J. (2021). Decolonizing health care: Challenges of cultural and epistemic pluralism in medical decision-making with indigenous communities. Bioethics, 35(8), 767–778. 10.1111/bioe.12946 34551134

[bibr25-13634615241296301] Cohen G. A. (2012). Finding oneself in the other. Princeton University Press.

[bibr26-13634615241296301] Collins H. M. (2023). The most important thing about science is values. Interdisciplinary Science Reviews, 48(2), 264–275. 10.1080/03080188.2022.2150414

[bibr27-13634615241296301] Collins H. M. (2024). Establishing veritocracy: Society, truth, and science. Transcultural Psychiatry, 61(5), 783–794. https://doi.org/10.1177/1363461524126072 10.1177/13634615241260726 PMC11629590 38863344

[bibr28-13634615241296301] Collins H. M. Evans R. (2017). Why democracies need science. John Wiley & Sons.

[bibr29-13634615241296301] Collins H. M. Evans R. Durant D. Weinel M. (2020). Experts and the Will of the People. *Palgrave Macmillan*.

[bibr30-13634615241296301] Collins H. M. Pinch T. (1998). The Golem: What you should know about science. Cambridge University Press.

[bibr31-13634615241296301] Collins H. M. Pinch T. (2008). Dr. Golem: How to think about medicine. University of Chicago Press.

[bibr32-13634615241296301] Connors M. H. Halligan P. W. (2017). Belief and belief formation: Insights from delusions. In Angel H. F. Oviedo L. Paloutzian R. F. Runehov A. L. Seitz R. J. (Eds.), Processes of believing: The acquisition, maintenance, and change in creditions (pp. 153–165). Springer.

[bibr33-13634615241296301] Corpuz J. C. G. (2021). Adapting to the culture of ‘new normal’: An emerging response to COVID-19. Journal of Public Health, 43(2), e344–e345. 10.1093/pubmed/fdab057 PMC7989434 33683346

[bibr34-13634615241296301] Cosentino G. (2020). Social media and the post-truth world order: the global dynamics of disinformation. Springer Nature.

[bibr35-13634615241296301] Covitt B. A. Anderson C. W. (2022). Untangling trustworthiness and uncertainty in science: Implications for science education. Science & Education, 31(5), 1155–1180. 10.1007/s11191-022-00322-6 35136284 PMC8815018

[bibr36-13634615241296301] Crichton P. Carel H. Kidd I. J. (2017). Epistemic injustice in psychiatry. BJPsych Bulletin, 41(2), 65–70. 10.1192/pb.bp.115.050682 28400962 PMC5376720

[bibr37-13634615241296301] Curtis V. (2013). Don't look, don't touch: The science behind revulsion. OUP Oxford.

[bibr38-13634615241296301] Curtis V. de Barra M. (2018). The structure and function of pathogen disgust. Philosophical Transactions of the Royal Society B: Biological Sciences, 373(1751), 20170208. 10.1098/rstb.2017.0208 PMC6000136 29866921

[bibr39-13634615241296301] Davies M. Coltheart M. (2000). Introduction: Pathologies of belief. Mind & Language, 15(1), 1–46. 10.1111/1468-0017.00122

[bibr40-13634615241296301] Douglas K. M. Sutton R. M. (2023). What are conspiracy theories? A definitional approach to their correlates, consequences, and communication. Annual Review of Psychology, 74, 271–298. 10.1146/annurev-psych-032420-031329 36170672

[bibr41-13634615241296301] Elshakry M. (2010). When science became western: Historiographical reflections. Isis, 101(1), 98–109. 10.1086/652691 20575492

[bibr42-13634615241296301] Festinger L. (1956). When Prophecy Fails. University of Minnesota Press.

[bibr43-13634615241296301] Fiedler K. Wänke M. (2009). The cognitive-ecological approach to rationality in social psychology. Social Cognition, 27(5), 699–732. 10.1521/soco.2009.27.5.699

[bibr44-13634615241296301] Foucault M. (2007). The Politics of Truth. Semiotext (e).

[bibr45-13634615241296301] Freeman D. Waite F. Rosebrock L. Petit A. Causier C. East A. Lambe S. (2022). Coronavirus conspiracy beliefs, mistrust, and compliance with government guidelines in England. Psychological Medicine, 52(2), 251–263. 10.1017/S0033291720001890 32436485 PMC7264452

[bibr46-13634615241296301] Fricker M. Graham P. J. Henderson D. Pedersen N. J., & Wyatt, J. (Eds.) (2020). The Routledge handbook of social epistemology. Routledge.

[bibr47-13634615241296301] Gabarron E. Oyeyemi S. O. Wynn R. (2021). COVID-19-related misinformation on social media: A systematic review. Bulletin of the World Health Organization, 99(6), 455. 10.2471/BLT.20.276782 34108756 PMC8164188

[bibr48-13634615241296301] Galbraith N. (2021). Delusions and pathologies of belief: Making sense of conspiracy beliefs via the psychosis continuum. In Cardella V. Gangemi A. (Eds.), Psychopathology and philosophy of mind: What mental disorders can tell US about our minds (pp. 117–144). Routledge.

[bibr49-13634615241296301] Galison P. Proctor R. (2020). Agnotology in action: A dialogue. In Kourany J. Carrier M. (Eds.), Science and the production of ignorance: When the quest for knowledge is thwarted. MIT Press.

[bibr50-13634615241296301] Gellner E. (2013). Postmodernism, reason and religion. Routledge.

[bibr51-13634615241296301] Ghai A. Sabour E. Salonga R. Ho R. Apollonio D. E. (2024). Exposures to bleach, peroxide, disinfectants, antimalarials, and ivermectin reported to the California poison control system before and during the COVID-19 pandemic, 2015–2021. Public Health Reports, 139(1), 112–119. 10.1177/00333549231201679 37933467 PMC10905766

[bibr52-13634615241296301] Gigerenzer G. (2020). What is bounded rationality? In Routledge handbook of bounded rationality (pp. 55–69). Routledge.

[bibr53-13634615241296301] Gold J. Gold I. (2015). Suspicious minds: How culture shapes madness. Simon and Schuster.

[bibr54-13634615241296301] Goldman A. I. (1987). Foundations of social epistemics. Synthese, 73(1), 109–144. 10.1007/BF00485444

[bibr55-13634615241296301] Goldman A. Whitcomb D. 2011). Social epistemology: essential readings. Oxford University Press.

[bibr56-13634615241296301] Good B. J. (1993). Medicine, rationality and experience: an anthropological perspective. Cambridge University Press.

[bibr57-13634615241296301] Greenspan R. L. Loftus E. F. (2021). Pandemics and infodemics: Research on the effects of misinformation on memory. Human Behavior and Emerging Technologies, 3(1), 8–12. 10.1002/hbe2.228 33363274 PMC7753404

[bibr58-13634615241296301] Habermas J. (2023). A new structural transformation of the public sphere and deliberative politics. John Wiley & Sons.

[bibr59-13634615241296301] Han B. C. (2018). What is power? John Wiley & Sons.

[bibr60-13634615241296301] Hari J. (2022). Stolen Focus: Why You Can't Pay Attention. Bloomsbury Publishing.

[bibr61-13634615241296301] Henrich J. Albers W. Boyd R. Gigerenzer G. McCabe K. A. Ockenfels A. Young H. P. (2001). What is the role of culture in bounded rationality? In Gigerenzer G. Selten R. (Eds.), Bounded rationality: The adaptive toolbox. Dahlem workshop report (pp. 343–359). MIT Press.

[bibr62-13634615241296301] Henschke A. Sussex M. O’Connor C. (2020). Countering foreign interference: Election integrity lessons for liberal democracies. Journal of Cyber Policy, 5(2), 180–198. 10.1080/23738871.2020.1797136

[bibr63-13634615241296301] Hofstadter R. (1965). The Paranoid Style in American Politics and Other Essays. Vintage Books.

[bibr64-13634615241296301] Ibrahim N. K. (2020). Epidemiologic surveillance for controlling COVID-19 pandemic: Types, challenges and implications. Journal of Infection and Public Health, 13(11), 1630–1638. 10.1016/j.jiph.2020.07.019 32855090 PMC7441991

[bibr65-13634615241296301] Kahneman D. (2011). Thinking, fast and slow. Macmillan.

[bibr66-13634615241296301] Kam C. D. (2019). Infectious disease, disgust, and imagining the other. The Journal of Politics, 81(4), 1371–1387. 10.1086/704438

[bibr67-13634615241296301] Kelly T. (2002). The rationality of belief and some other propositional attitudes. Philosophical Studies, 110, 163–196. 10.1023/A:1020212716425

[bibr68-13634615241296301] Kirchhoff M. D. Kiverstein J. (2019). Extended consciousness and predictive processing: A third-wave view. Routledge.

[bibr69-13634615241296301] Kirmayer L. J. (1983). Paranoia and pronoia: The visionary and the banal. Social Problems, 31(2), 170–179. 10.2307/800208

[bibr70-13634615241296301] Kirmayer L. J. (1990). Resistance, reactance, and reluctance to change: A cognitive attributional approach to strategic interventions. Journal of Cognitive Psychotherapy, 4(2), 83. 10.1891/0889-8391.4.2.83

[bibr71-13634615241296301] Kirmayer L. J. (2012). Cultural competence and evidence-based practice in mental health: Epistemic communities and the politics of pluralism. Social Science & Medicine, 75(2), 249–256. 10.1016/j.socscimed.2012.03.018 22575699

[bibr72-13634615241296301] Kirmayer L. J. Ramstead M. J. (2017). Embodiment and enactment in cultural psychiatry. In Tewes C. Durt C. Fuchs T. (Eds.), Embodiment, enaction, and culture: Investigating the constitution of the shared world (pp. 397–422). MIT Press.

[bibr73-13634615241296301] Klintman M. (2022). Knowledge resistance. In Gross M. McGoey L. (Eds.), Routledge international handbook of ignorance studies (pp. 323–333). Routledge. (2015).

[bibr74-13634615241296301] Knorr-Cetina K. (2007). Culture in global knowledge societies: Knowledge cultures and epistemic cultures. Interdisciplinary Science Reviews, 32(4), 361–375. 10.1179/030801807X163571

[bibr75-13634615241296301] Korzybski A. (1941). General semantics, psychiatry, psychotherapy and prevention. American Journal of Psychiatry, 98(2), 203–214. 10.1176/ajp.98.2.203

[bibr76-13634615241296301] Korzybski A. (1958). Science and sanity: An introduction to non-Aristotelian systems and general semantics. Institute of General Semantics.

[bibr77-13634615241296301] The Lancet Infectious Diseases (2020). [Editorial] the COVID-19 infodemic. The Lancet Infectious Diseases, 20(8), 875. 10.1016/S1473-3099(20)30565-X 32687807 PMC7367666

[bibr78-13634615241296301] Lapsley D. Chaloner D. (2020). Post-truth and science identity: A virtue-based approach to science education. Educational Psychologist, 55(3), 132–143. 10.1080/00461520.2020.1778480

[bibr79-13634615241296301] La Rocca G. Carignan M. E. Artieri G. B. (eds) (2023). Infodemic Disorder: COVID-19 Coping Strategies in Europe, Canada and Mexico. Springer Nature.

[bibr80-13634615241296301] Laudan L. (2012). Science and Relativism. University of Chicago Press.

[bibr81-13634615241296301] Levy N. (2023). Echoes of covid misinformation. Philosophical Psychology, 36(5), 931–948.

[bibr82-13634615241296301] Manski C. F. Molinari F. (2021). Estimating the COVID-19 infection rate: Anatomy of an inference problem. Journal of Econometrics, 220(1), 181–192. 10.1016/j.jeconom.2020.04.041 32377030 PMC7200382

[bibr83-13634615241296301] McHugh N. A. (2017). Epistemic communities and institutions. In Kidd I. J. Medina J. Polhaus G. (Eds.), The Routledge handbook of epistemic injustice (pp. 270–278). Routledge.

[bibr84-13634615241296301] McLuhan M. (1964). Understanding media: The extensions of man. Routledge.

[bibr85-13634615241296301] Michaels D. (2006). Manufactured uncertainty: Protecting public health in the age of contested science and product defense. Annals of the New York Academy of Sciences, 1076(1), 149–162. 10.1196/annals.1371.058 17119200

[bibr86-13634615241296301] Milanovic B. (2016). Global inequality. Harvard University Press.

[bibr87-13634615241296301] Nattrass N. (2023). Promoting conspiracy theory: From AIDS to COVID-19. Global Public Health, 18(1), 2172199. 10.1080/17441692.2023.2172199 36749932

[bibr88-13634615241296301] Navin M. (2013). Competing epistemic spaces: How social epistemology helps explain and evaluate vaccine denialism. Social Theory and Practice, 39 (2), 241–264. 10.5840/soctheorpract201339214

[bibr89-13634615241296301] Nguyen C. T. (2020). Echo chambers and epistemic bubbles. Episteme, 17(2), 141–161. https://doi.org/10.1017/epi.2018.32

[bibr90-13634615241296301] Niiniluoto I. (2020). Social aspects of scientific knowledge. Synthese, 197(1), 447–468. 10.1007/s11229-018-1868-7

[bibr91-13634615241296301] Nilsson N. J. (2014). Understanding beliefs. MIT Press.

[bibr92-13634615241296301] Obejas A. (2021). Boomerang. Beacon Press.

[bibr93-13634615241296301] O’Connor C. Weatherall J. O. (2019). The misinformation age. Yale University Press.

[bibr94-13634615241296301] Olaniran B. Williams I. (2020). Social media effects: Hijacking democracy and civility in civic engagement. In Jones J. Trice M. (Eds.), Platforms, protests, and the challenge of networked democracy. Rhetoric, politics and society (pp. 77–94). Palgrave Macmillan. https://doi.org/10.1007/978-3-030-36525-7_5

[bibr95-13634615241296301] O’Neil C. (2016). Weapons of math destruction: How big data increases inequality and threatens democracy. Broadway Books.

[bibr96-13634615241296301] Oreskes N. (2019). Why trust science? Princeton University Press.

[bibr97-13634615241296301] Oreskes N. Conway E. M. (2008). Challenging knowledge: How climate science became a victim of the cold war. In Proctor R. N. Schiebinger L. (Eds.), Agnotology: The making and unmaking of ignorance (pp. 55–89). Stanford University Press.

[bibr98-13634615241296301] Parr T. Pezzulo G. Friston K. J. (2022). Active inference: the free energy principle in mind, brain, and behavior. MIT Press.

[bibr99-13634615241296301] Perlis R. H. Trujillo K. L. Green J. Safarpour A. Druckman J. N. Santillana M. Lazer D. (2023). Misinformation, trust, and use of ivermectin and hydroxychloroquine for COVID-19. JAMA Health Forum, 4(9), e233257. 10.1001/jamahealthforum.2023.3257 PMC10542734 37773507

[bibr100-13634615241296301] Pezzulo G. Rigoli F. Friston K. (2015). Active inference, homeostatic regulation and adaptive behavioural control. Progress in Neurobiology, 134, 17–35. 10.1016/j.pneurobio.2015.09.001 26365173 PMC4779150

[bibr101-13634615241296301] Pigliucci M. (2015). Scientism and pseudoscience: A philosophical commentary. Journal of Bioethical Inquiry, 12(4), 569–575. 10.1007/s11673-015-9665-1 26615544

[bibr102-13634615241296301] Pigliucci M. Boudry M. 2013). Philosophy of pseudoscience: Reconsidering the demarcation problem. University of Chicago Press.

[bibr103-13634615241296301] Pilkington E. (2020). Revealed: Leader of group peddling bleach as coronavirus ‘cure’ wrote to Trump this week. The Guardian, April 24, 2020. https://www.theguardian.com/world/2020/apr/24/revealed-leader-group-peddling-bleach-cure-lobbied-trump-coronavirus

[bibr104-13634615241296301] Pothos E. M. Busemeyer J. R. (2022). Quantum cognition. Annual Review of Psychology, 73(1), 749–778. 10.1146/annurev-psych-033020-123501 34546804

[bibr105-13634615241296301] Proctor R. N. (2008). Agnotology: A missing term to describe the cultural production of ignorance (and its study). In Proctor R. N. Schiebinger L. (Eds.), Agnotology: The making and unmaking of ignorance (pp. 1–33). Stanford University Press.

[bibr106-13634615241296301] Pynchon T. (1973). Gravity's rainbow. Viking.

[bibr107-13634615241296301] Ramstead M. J. Veissière S. P. Kirmayer L. J. (2016). Cultural affordances: Scaffolding local worlds through shared intentionality and regimes of attention. Frontiers in Psychology, 7, 1090. 10.3389/fpsyg.2016.01090 27507953 PMC4960915

[bibr108-13634615241296301] R’boul H. (2022). Postcolonial interventions in intercultural communication knowledge: Meta-intercultural ontologies, decolonial knowledges and epistemological polylogue. Journal of International and Intercultural Communication, 15(1), 75–93. 10.1080/17513057.2020.1829676

[bibr109-13634615241296301] Reiter B. (2020). Fuzzy epistemology: Decolonizing the social sciences. Journal for the Theory of Social Behaviour, 50(1), 103–118. 10.1111/jtsb.12229

[bibr110-13634615241296301] Rose J. (2020). To believe or not to believe: An epistemic exploration of fake news, truth, and the limits of knowing. Postdigital Science and Education, 2(1), 202–216. 10.1007/s42438-019-00068-5

[bibr111-13634615241296301] Rose L. Bartoli T. (2020). Agnotology and the epistemology of ignorance: A framework for the propagation of ignorance as a consequence of technology in a Balkanized media ecosystem. Postdigital Science and Education, 2(1), 184–201. 10.1007/s42438-019-00084-5

[bibr112-13634615241296301] Russell T. W. Golding N. Hellewell J. Abbott S. Wright L. Pearson C. A. Kucharski A. J. (2020). Reconstructing the early global dynamics of under-ascertained COVID-19 cases and infections. BMC Medicine, 18(1), 332. 10.1186/s12916-020-01790-9 33087179 PMC7577796

[bibr113-13634615241296301] Sadegh-Zadeh K. (2000). Fuzzy health, illness, and disease. The Journal of Medicine and Philosophy, 25(5), 605–638. 10.1076/0360-5310(200010)25:5;1-W;FT605 11035544

[bibr114-13634615241296301] Sanders S. (1975). Pynchon's paranoid history. Twentieth Century Literature, 21(2), 177–192. 10.2307/440707

[bibr115-13634615241296301] Siegert B. (2011). The map is the territory. Radical Philosophy, 169(Series 1), 13–16. https://www.radicalphilosophyarchive.com/issue-files/rp169_article3_mapistheterritory_siegert.pdf

[bibr116-13634615241296301] Simon F. M. Camargo C. Q. (2023). Autopsy of a metaphor: The origins, use and blind spots of the ‘infodemic’. New Media & Society, 25(8), 2219–2240. 10.1177/14614448211031908

[bibr117-13634615241296301] Sperber D. (1985). Anthropology and psychology: Towards an epidemiology of representations. Man, *20*(1), 73–89. 10.2307/2802222

[bibr118-13634615241296301] Stein R. A. Ometa O. Shetty S. P. Katz A. Popitiu M. I. Brotherton R. (2021). Conspiracy theories in the era of COVID-19: A tale of two pandemics. International Journal of Clinical Practice, 75(2). 10.1111/ijcp.13778 PMC7995222 33480171

[bibr119-13634615241296301] Sturm T. Albrecht T. (2021). Constituent COVID-19 apocalypses: Contagious conspiracism, 5G, and viral vaccinations. Anthropology & Medicine, 28(1), 122–139. 10.1080/13648470.2020.1833684 33233926

[bibr120-13634615241296301] Sunstein C. R. (2021). This is not normal: The politics of everyday expectations. Yale University Press.

[bibr121-13634615241296301] Tasnim S. Hossain M. M. Mazumder H. (2020). Impact of rumors and misinformation on COVID-19 in social media. Journal of Preventive Medicine and Public Health, 53(3), 171–174. 10.3961/jpmph.20.094 32498140 PMC7280809

[bibr122-13634615241296301] Van Dongen J. Paul H. (2017). Introduction: Epistemic virtues in the sciences and the humanities. In Van Dongen J. Paul H. (Eds.), Epistemic virtues in the sciences and the humanities (pp. 1–10). Springer International Publishing.

[bibr123-13634615241296301] Veissière S. P. Constant A. Ramstead M. J. Friston K. J. Kirmayer L. J. (2020). Thinking through other minds: A variational approach to cognition and culture. Behavioral and Brain Sciences, 43, e90. https://doi.org/10.1017/ S0140525X19001213 10.1017/S0140525X19001213 31142395

[bibr124-13634615241296301] Wang Y. McKee M. Torbica A. Stuckler D. (2019). Systematic literature review on the spread of health-related misinformation on social media. Social Science & Medicine, 240, 112552. 10.1016/j.socscimed.2019.112552 31561111 PMC7117034

[bibr125-13634615241296301] Young A. (1981). When rational men fall sick: An inquiry into some assumptions made by medical anthropologists. Culture, Medicine and Psychiatry, 5(4), 317–335. 10.1007/BF00054773 7326949

[bibr126-13634615241296301] Young A. (1982). Rational men and the explanatory model approach. Culture, Medicine and Psychiatry, 6(1), 57–71. 10.1007/BF00049471 7105790

[bibr127-13634615241296301] Zielinski C. (2021). Infodemics and infodemiology: A short history, a long future. Revista Panamericana de Salud Publica, 45, e40. 10.26633/RPSP.2021.40 PMC8110882 33995517

